# Estimating upper-extremity function from kinematics in stroke patients following goal-oriented computer-based training

**DOI:** 10.1186/s12984-021-00971-8

**Published:** 2021-12-31

**Authors:** Belén Rubio Ballester, Fabrizio Antenucci, Martina Maier, Anthony C. C. Coolen, Paul F. M. J. Verschure

**Affiliations:** 1 Laboratory of Synthetic, Perceptive, Emotive and Cognitive Systems (SPECS), Institute for Bioengineering of Catalonia (IBEC), The Barcelona Institute of Science and Technology (BIST), Baldiri Reixac 10-12, 08028 Barcelona, Spain; 2 Saddle Point Science Ltd, 10 Lincoln Street, York, UK; 3 Institució Catalana de Recerca, Estudis Avançats (ICREA), Barcelona, Spain

**Keywords:** Rehabilitation, Stroke, Interactive feedback, Upper extremities, Posture monitoring, Motion sensing, Motion classification, Multivariate regression

## Abstract

**Introduction:**

After a stroke, a wide range of deficits can occur with varying onset latencies. As a result, assessing impairment and recovery are enormous challenges in neurorehabilitation. Although several clinical scales are generally accepted, they are time-consuming, show high inter-rater variability, have low ecological validity, and are vulnerable to biases introduced by compensatory movements and action modifications. Alternative methods need to be developed for efficient and objective assessment. In this study, we explore the potential of computer-based body tracking systems and classification tools to estimate the motor impairment of the more affected arm in stroke patients.

**Methods:**

We present a method for estimating clinical scores from movement parameters that are extracted from kinematic data recorded during unsupervised computer-based rehabilitation sessions. We identify a number of kinematic descriptors that characterise the patients’ hemiparesis (e.g., movement smoothness, work area), we implement a double-noise model and perform a multivariate regression using clinical data from 98 stroke patients who completed a total of 191 sessions with RGS.

**Results:**

Our results reveal a new digital biomarker of arm function, the Total Goal-Directed Movement (TGDM), which relates to the patients work area during the execution of goal-oriented reaching movements. The model’s performance to estimate FM-UE scores reaches an accuracy of $R^2$: 0.38 with an error ($\sigma$: 12.8). Next, we evaluate its reliability ($r=0.89$ for test-retest), longitudinal external validity ($95\%$ true positive rate), sensitivity, and generalisation to other tasks that involve planar reaching movements ($R^2$: 0.39). The model achieves comparable accuracy also for the Chedoke Arm and Hand Activity Inventory ($R^2$: 0.40) and Barthel Index ($R^2$: 0.35).

**Conclusions:**

Our results highlight the clinical value of kinematic data collected during unsupervised goal-oriented motor training with the RGS combined with data science techniques, and provide new insight into factors underlying recovery and its biomarkers.

## Introduction

Stroke is the second major cause of death and disability worldwide, with about 15 million new cases every year [1]. One-third of these cases lead to persistent cognitive and motor disabilities [2]. About 80% of stroke survivors present weakness and partial loss of voluntary control in the upper-extremities [3], or hemiparesis, which is often associated with other sensorimotor alterations, such as hypertonia or tremor.

Although hemiparesis is a highly prevalent symptom and severely limits the independence of affected patients, its causes and recovery dynamics are not fully understood [4]. Recent literature converges on the idea that recovery is mainly due to a combination of residual corticospinal tract capacity and an upregulation of the reticulospinal tract [5, 6]. Further, recovery seems to follow a temporal structure where most of the improvement occurs during the first months post-stroke [7, 8]. So far the assessment of the hemiparesis phenotype and its progression, however, are based on assessment methods with known limitations (e.g. Fugl-Meyer Assessment [9, 10], Action Research Arm Test [11, 12]) and there is a need for more sensitive, objective, and reliable alternatives that are also compatible with contemporary digital health technologies.

A recent systematic review [13] of a total of 225 studies (N = 6197) using 151 different kinematic metrics found that kinematic assessments of upper limb sensorimotor function are poorly standardised and rarely measure clinimetrics in an unbiased manner. Specifically, using descriptors of accuracy, efficacy, efficiency, movement planning, precision, spatial posture, speed, temporal posture, and range of movement together with clinimetric properties of these descriptors (i.e., reliability, measurement error, convergent validity, and external validity), the authors showed that the studies analysed exclusively focused on finding correlations between measures of impairment, and only two of the studies reported correlations in change. Overall, there is very limited information regarding test-retest reliability and the external validity of the change of kinematic outcome measures of reaching performance [14]. Exceptionally, Murphy et al. [15] explored external validity of the change in a number of kinematic descriptors and found a significant covariation of the Action Research Arm Test (ARAT) scores with movement time ($R^2$ = 0.36), smoothness ($R^2$ = 0.31), and trunk displacement ($R^2$ = 0.35). Although the results are promising, this study involves a limited number of subjects (N = 24) from a highly homogeneous sample (i.e., acute patients only). Further, the ARAT clinical scale presents poor robustness to compensation and is especially vulnerable to the use of explicit strategies to improve performance. Majeed et al. [16] explored the application of models based on LASSO regression to predict changes in motor ability (FM-UE) and motor function (Wolf Motor Function Test, WMFT). These models proposed that recovery in both scales can be approximated by the patient’s age, the patient’s motor control during the execution of fast movements, and other demographic and clinical features, altogether accounting for 65% and 86% of the variability for the FM-UE and WMFT scales respectively. Although these models reached exceptional accuracy, their utility is limited because they make use of kinematic data obtained during the execution of very specific pointing movements supervised by clinicians and/or researchers, and are based on generic unbounded linear models, with the consequence that their estimated values could be largely outside the meaningful range of the scale.

We propose a new approach towards using kinematic data obtained in unsupervised rehabilitation sessions to estimate the level of impairment and functional recovery. Data is obtained from patients engaging with goal-oriented embodied individualised training with the Rehabilitation Gaming System (RGS) [17, 18]. The RGS combines the paradigm of action execution with that of observation of the corresponding movement in Virtual Reality (VR), this goal is achieved by having the patients perform tasks from a first-person perspective, where the movement of their limbs are captured by a camera or a depth sensor (i.e. Microsoft Kinect) and mapped to an analogous virtual representation on a computer screen. RGS includes individualisation mechanisms to adjust the difficulty of the task to the capabilities of the patient, contextual restrictions, and explicit and implicit feedback.

We first explore the potential of hand movements collected during unsupervised RGS sessions to characterise hemiparesis in stroke patients. Secondly, we build and analyse the performance (i.e., test-retest reliability, validity, sensitivity, and generalisation) [13] of a model for estimating impairment and recovery scores captured by standardised clinical scales.

## Methods

### Subjects

Our retrospective analysis uses data of 191 RGS sessions from 98 individuals with hemiparesis (age in [23, 87], mean 63; days post-stroke in [5, 3045], mean 400; Table 1) who were recruited between 2010 and 2015 to participate in studies conducted in Barcelona and Tarragona, Spain [18–20]. Participants met the following inclusion criteria: (1) ischemic strokes (middle cerebral artery territory) or hemorrhagic strokes (intracerebral); (2) mild-to-moderate upper limb hemiparesis (Medical Research Council scale for proximal muscles $>2$) after a first-ever stroke; (3) age between 20 and 90 years old; and (4) the absence of any significant cognitive impairment (Mini-Mental State Evaluation $>22$).

**Table 1:** Characteristics of the 191 samples composing the main dataset (single session, *Spheroids* scenario)

Variables	Range [min, max]	Mean	SD	*r*(FM-UE)	*r*(CAHAI)	*r*(BI)	Missing
FM-UE score	[4, 66]	43	16	1	0.89	0.34	–
CAHAI score	[13, 91]	52	26	0.89	1	0.50	–
BI score	[10, 100]	80	21	0.34	0.50	1	15
1. Gender	Female/male	73/118	–	0.17	0.11	(**0.036**)	–
2. Age	[23, 87]	63	12.8	(− **0.015**)	− 0.10	− 0.30	–
3. Dominant side more affected	Yes/no	72/119	–	(− **0.013**)	(**0.039**)	0.21	–
4. Time since stroke (days)	[5, 3045]	400	625	− 0.25	− 0.17	0.22	–
5. Sessions completed so far	[2, 49]	10	11	0.31	0.38	0.32	–
*6. Work area (*$m^2$*)*	[0.011, 1.8]	0.38	0.35	0.29	0.27	0.14	–
*7. Distance covered(m)*	[2.6, 240]	56	34	0.18	0.21	0.26	-
*8. Performance (% success)*	[0.37, 0.94]	0.68	0.105	0.33	0.37	0.33	-
*9. Maximum reaching speed (m*/*s)*	[2.8, 88]	18	16	0.17	0.13	(**0.061**)	–
*10. Difficulty level reached*	[− 0.16, 0.89]	0.46	0.23	0.45	0.52	0.46	–
*11. Smoothness (mm)*	[0.17, 3.7]	1.2	0.55	0.42	0.39	0.28	–
*12. TGDM (m)*	[0.011, 0.12]	0.062	0.021	0.52	0.58	0.43	–

The *r* columns refer to the Pearson correlation coefficients with the FM-UE, CAHAI, and BI clinical scales, respectively. Correlations below the threshold r $\sim 0.081$ (Fig. 12 in Appendix) are in parenthesis. The clinical scales are measured no more than 4 days before or after the coupled RGS session. The variables (6–12) are obtained directly from RGS log files. Characteristics of second-order variables for this dataset are shown in Table 5 in Appendix

### Protocol

**Fig. 1:**
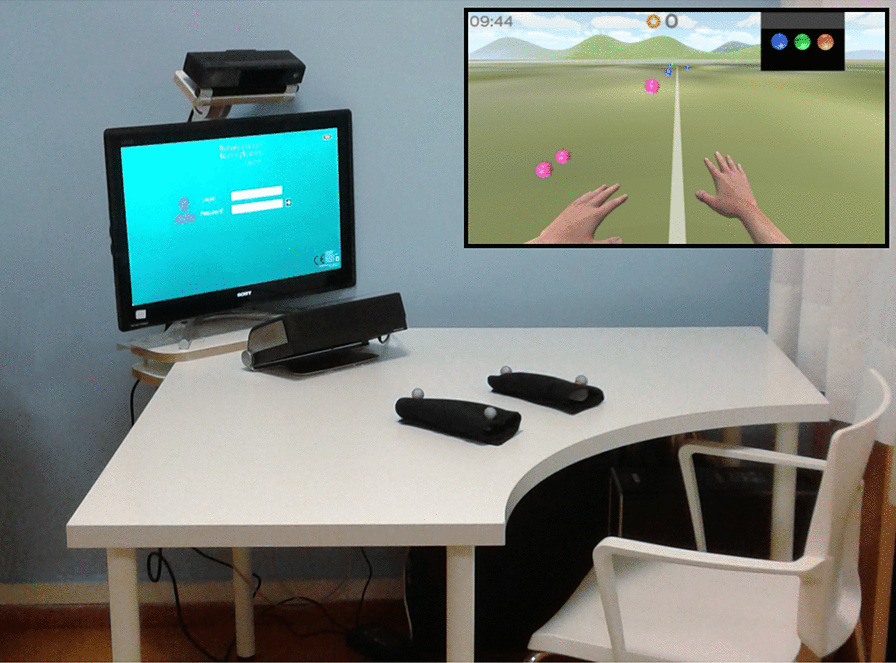
The RGS system [17, 18] used in the rehabilitation protocol followed by the patients in the collection of the data in this study. In the inset, we show a screenshot from the ‘Spheroids’ activity during an RGS session

Participants followed a rehabilitation protocol including 3–5 weekly sessions of 30 minutes each for 3–12 weeks using the RGS in Fig. 1. The RGS consists of a PC, a 17 inch LCD touch display, an image-based motion capture device positioned on top of the screen [18]. The virtual tasks logic and graphics are implemented using the Torque 3D and Unity 3D game engines. The joint movements of the user’s head, shoulders and elbows are tracked and mapped onto an avatar through a biomechanical model using a custom-developed vision-based motion capture system. Arm movements are displayed on a screen from a first-person perspective, realising a rehabilitation paradigm that combines goal-oriented embodied action execution and observation.

For the RGS sessions in the main dataset (Table 1) the participants are instructed to intercept virtual spheres by executing horizontal bimanual movements over the surface of a table (‘Spheroids’ protocol, cf. inset in Fig. 1). The task parameters (the frequency of sphere appearance, their speed, their range, and size) are combined in a single parameter (‘difficulty level’) and automatically adjusted during the session in order to maintain the user’s performance between 70 and 80% success rate [17, 21]. The system allows for the storage and extraction of performance parameters as well as hand path trajectories derived from joints’ positions and rotations recorded at about 100 Hz.

During the rehabilitation patients are evaluated using standard clinical scales: Fugl-Meyer Assessment for the upper extremity (FM-UE), Chedoke Arm and Hand Activity Inventory (CAHAI) and Barthel Index (BI). When collecting the 191 samples (Table 1), the following measures are taken to improve data quality:

- The clinical score measurements (FM-UE, CAHAI, BI) are coupled to the RGS session closest in time, with a maximum time separation of 4 days between the measurement and the RGS session;
- The first two RGS sessions of a patient at the start of the rehabilitation trajectory are excluded to ensure that patients are familiar with the RGS environment for all collected samples.

### Outcomes and analysis

To analyse the potential of RGS-derived movement descriptors for capturing both impairment and recovery in standardised clinical scales, we first extract a set of variables that are known to correlate with the severity of hemiparesis [13, 16]. Next, we define a model that combines the information of several variables to estimate the patient’s score on the FM-UE clinical scale. The model includes an error estimate. We use repeated cross-validation to avoid overfitting, allocating 50% of the samples to the training set and 50% to the validation set. We study the model evaluating its convergent validity, test-retest reliability, external validity, and generalisation to other tasks and clinical scales.

#### Analysis of convergent validity: estimating impairment

To explore the convergent validity of RGS-derived kinematic descriptors in comparison to standardised clinical assessments, we compute Pearson correlations between all the variables (the RGS-derived descriptors and the baseline characteristics) and the clinical scores. By comparing these to a randomised outcome distribution we identify a threshold of $r \simeq 0.081$ for all the Pearson correlations variable-scores (Fig. 12 in Appendix). Subsequently, we adopt a nonlinear parametric model to combine information from several variables for estimating the patients’ impairment level (i.e., FM-UE scale). We use only the patient’s baseline characteristics and RGS-derived movement descriptors (extracted from a single session logfile) as predictors. In particular, we rule out the two variables *sessions completed so far* and *time since stroke* and functions of them. In this way, the estimate is intended to assess the clinical status of the patient at a given moment without knowledge of the rehabilitation history. To avoid overfitting, we perform repeated cross-validation with 50% of samples for training and 50% for validation, obtaining the optimal active set of variables possible for our dataset. In the cross-validation procedure, we define the accuracy of the model as the percentage of samples that are correctly estimated above or below the median score (e.g. 47 for FM-UE). Additionally, we report the values of the Leave-one-out cross-validation (LOOCV) for the optimal active set. To quantify the performance of the model, we compare true and estimated FM-UE scores reporting the average error, the Pearson correlation, and the coefficient of determination. Finally, we report the performance of the estimation of FM-UE obtained by a linear model with the same active set of variables.

#### Analysis of test–retest reliability

To evaluate the test-retest reliability, we consider two unseen datasets each composed of 921 RGS sessions, for a total of 1842 unseen RGS sessions. Each session in the first dataset ‘test’ is associated with a session in the second dataset ‘retest’ and obtained from the same patient within less than 48 h. The small time frame makes it plausible that the clinical state of the patient is unchanged between the two test–retest sessions, and so they can be used to assess the reliability of the regression models. These data were collected in the same trials as the main dataset, but they correspond to rehabilitation sessions for which we do not have an associated measurement of a clinical scale (so they cannot be used for training the model). To quantify the reliability in the estimation of the FM-UE scores, we evaluate the intraclass correlation coefficient (ICC) between the estimations obtained by the model for test and retest sessions.

#### Analysis of external validity: estimating the change in impairment

Starting from the original dataset (*Spheroids*, Table 1), we design a new dataset (recovery dataset, Table 2) composed of 54 samples where each sample represents a couple of sessions of the same patient for which the time lapsed between the two is larger than 16 days. Next, we compute the correlations between the change in the movement descriptors and clinical improvements. We utilise this dataset to analyse the external validity of the previous model (obtained for estimating impairment) in detecting changes in the clinical status of the same patient. Therefore, we adopt the same set of variables used for the estimate of impairment. We evaluate the performance of the model by comparing the true and predicted change of FM-UE score. Next, we identify 38 out of the 54 samples from the recovery dataset (Table 2) for which the associated $\Delta {\text {FM-UE}}$ values exceed an MDC of 4 points. We then determine the sensitivity of the model evaluating the percentage of $\Delta {\text {FM-UE}}$ that are correctly predicted above the MDC.

**Table 2:** Characteristics of the 54 samples composing the recovery dataset

Variables	Range [min,max]	Mean	SD	$r(\Delta {\text {FM-UE}})$	$r(\Delta \text {CAHAI})$	$r(\Delta \text {BI})$	Missing
Change in FM-UE score	[− 2, 35]	9.1	9.1	1	0.68	0.67	–
Change in CAHAI score	[− 1, 75]	25	19	0.68	1	0.72	–
Change in BI score	[− 6, 69]	19	22	0.67	0.72	1	13
1. Gender	Female/male	24/30	–	(− **0.0046**)	0.14	0.29	–
2. Age	[42, 84]	65	13	− 0.25	(**0.061**)	(**0.051**)	-
3. Dominant side more affected	Yes/no	24/30	–	− 0.21	(**0.0065**)	(− **0.043**)	–
4. Days elapsed between the two sessions	[17,89]	49	26	(**0.037**)	0.31	0.39	
5. Sessions completed so far (at first)	[5, 46]	23	14	− 0.45	− 0.33	− 0.46	–
6. *Change in work area* ($m^2$)	[− 1.2, 1.2]	0.066	0.43	0.15	(**0.048**)	0.12	–
7. *Change in distance covered* (*m*)	[− 68, 75]	14	23	0.21	0.22	0.19	–
8. *Change in performance (% success)*	[− 0.17, 0.40]	0.076	0.11	(**0.075**)	(**0.12**)	0.22	–
9. *Change in max. reaching speed* (*m*/*s*)	[− 51, 77]	3.3	21	(**0.087**)	(**0.048**)	(**0.063**)	–
10. *Change in difficulty*	[− 0.12, 0.91]	0.21	0.20	0.38	0.39	0.36	–
11. *Change in smoothness* (*mm*)	[− 0.51, 1.8]	0.22	0.50	0.37	0.35	0.39	–
12. *Change in TGDM* (*m*)	[− 0.046, 0.14]	0.024	0.035	0.48	0.55	0.49	–
Initial FM score	[13,66]	44	15	− 0.44	− 0.20	(− **0.13**)	–
Initial CAHAI score	[14,90]	48	22	− 0.50	− 0.50	− 0.38	–
Initial BI score	[31,100]	72	23	− 0.60	− 0.64	− 0.82	13

We select couple of sessions of the same patient with a delay of at least 16 days from the main dataset, Table 1. The last three columns report the Pearson coefficient correlation between the variable and the change in the clinical score between the two sessions. Correlations below the threshold $r \sim 0.14$ are in parenthesis. Characteristics of second-order variables for this dataset are shown in Table 6 in Appendix

#### Analysis of task generalisation

The *Spheroids* protocol requires predominantly movements in the lateral (left/right) direction. To explore the potential of the model to generalise to other tasks that involve bimanual 2D planar reaching movements, we identify a second dataset of 37 samples from a previous study in which 19 subacute stroke patients with hemiparesis trained with a different RGS-based activity which is a variation on the well-known arcade game ‘Whac-A-Mole’ [18, 22]. The observed FM-UE scores in this dataset are in the range [5, 60], with a mean of 37 and an SD of 14. The gameplay of *Whac-A-Mole* requires movements on the full 2d plane so, besides the movement descriptors used for Spheroids, we define independent descriptors for the movements in the front/back direction. Subsequently, we adopt the nonlinear parametric model to combine information from different variables to estimate the impairment level as measured by the FM-UE scale. To quantify the performance of the model, we compare true and estimated FM-UE scores reporting the average error, the Pearson correlation, and the coefficient of determination.

#### Analysis of generalisation to other clinical scales

To explore the potential of the model to generalise for the estimation of impairment captured by other standardised clinical approaches, we considered the estimation of the CAHAI and BI scales that are available in the Spheroids dataset (Table 1). We, therefore, follow the same steps as above to analyse the convergent validity of the model in estimating CAHAI and BI scores. It is useful to anticipate here that the results for CAHAI are close to those for FM-UE. This similarity is expected as the two scales have a high relative correlations and comparable correlations to most of the variables, cf. Table 1. To be quantitative, we can consider the case in which the FM-UE score $S_i^{\text {FM}}$ of the ‘Spheroids’ dataset is estimated by simply rescaling the corresponding CAHAI value $S_i^{\text {CAHAI}}$ by 66/91; this leads to the standard error

1 $$\begin{aligned} \frac{1}{191}\sqrt{\sum _{i=1}^{191} \left[ \left( S_i^{\text {FM}} - (66/91) S_i^{\text {CAHAI}}\right) ^2\right] } \simeq 10.1 \end{aligned}$$

and an $R^2$ value of $1 - \sigma _{\text {FM,CAHAI}}^2 / \sigma ^2_{\text {FM}} \simeq 0.62$. Estimating BI scores from kinematic descriptors is instead generally harder. This can be explained by the lower correlation of BI scores with most of the variables (Table 1). If we estimate the FM-UE score by a simple rescaling of the BI value by 66/100 we get a standard error of

2 $$\begin{aligned} \frac{1}{176}\sqrt{\sum _{i=1}^{176} [(S_i^{\text {FM}} - (66/100) S_i^{\text {BI}})^2 ]} \simeq 15.5 \end{aligned}$$

and a $R^2$ value of $1 - \sigma _{\text {FM,BI}}^2 / \sigma ^2_{\text {FM}} \simeq 0.12$. So we see again that BI carries different information than FM-UE (or CAHAI). The above values give us a natural benchmark to assess the performance of the model estimation of the clinical scales.

We conducted these analyses using in-house software (SaddlePoint Signature v2.9.3).

### Identification of variables

Given the technical constraints of the setup (e.g., sampling rate) and the nature of the training protocol (i.e., execution of horizontal reaching movements), we extracted all task-relevant kinematic measures of arm use that we could identify in the literature [13] . In total we obtained 31 variables, 12 first-order variables and 19 second-order variables obtained as functions of the first-order ones.

We identify two groups of first-order variables: (1) demographic and physiological data at recruitment, variables 1–5 in Table 1, and (2) kinematic descriptors extracted for the more affected limb during training sessions, variables 6–12 in Table 1. For the evaluation of all kinematic descriptors, the first and last two minutes of each training session are discarded to avoid the interference of behaviours or events related to the start and the ending of the training session (i.e., revision of instructions, postural adjustments, exposure to the final score screen, etc.).

Second-order variables include chronicity (i.e. acute, sub-acute, and chronic categories) and the difference between the less and the more affected upper-limb in each of the quantitative first-order variables, as well as their logarithmic transformations. The descriptive statistics of second-order variables are given in Table 5 in the Appendix.

**Fig. 2:**
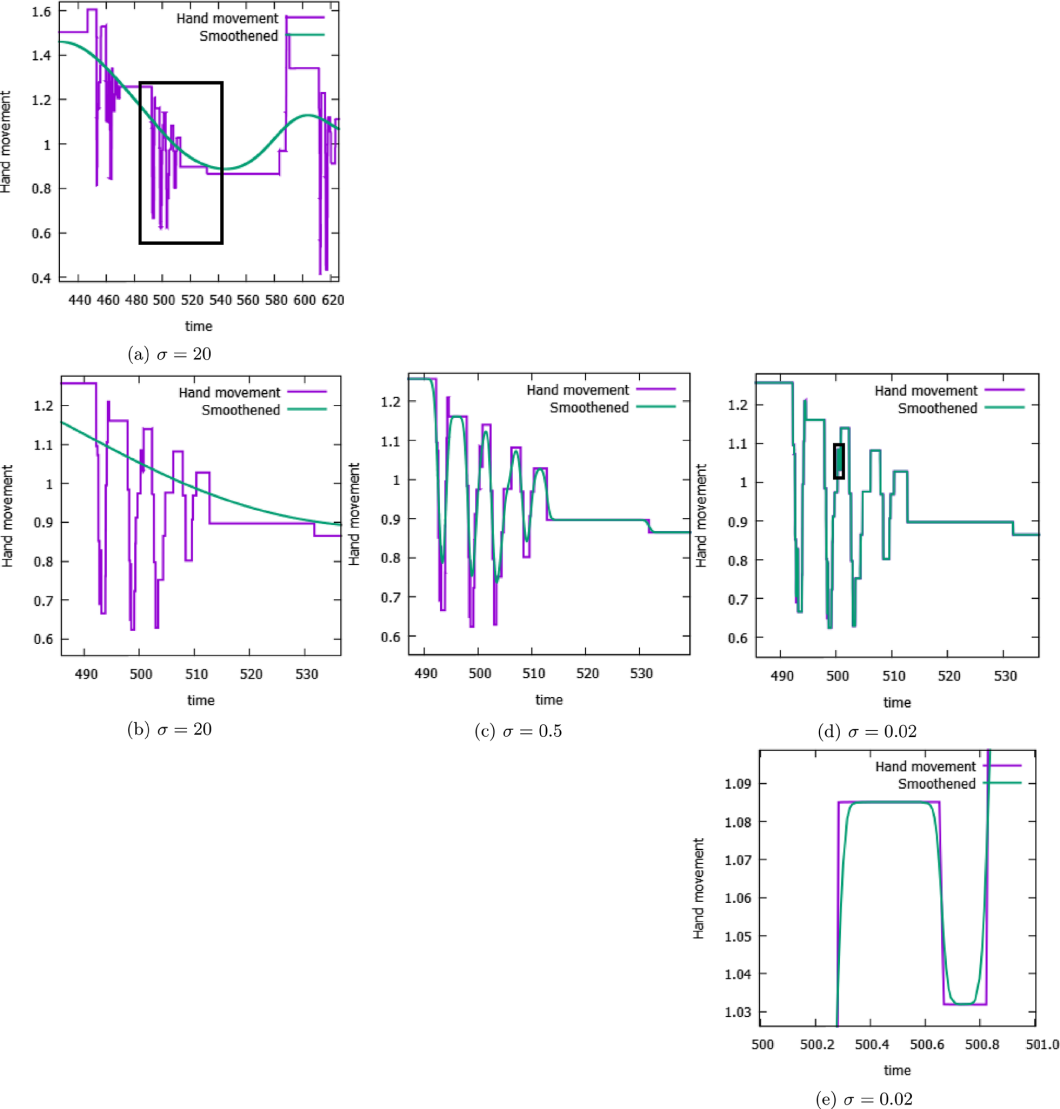
An example of the smoothed trajectory $f_\sigma (t)$ Eq. 3 (green line) for lateral (left/right) direction hand movements with $\sigma = 20 s$ (left) $\sigma = 0.5 s$ (center) and $\sigma = 0.02 s$ (right) compared to the real trajectory as recorded by the camera (purple line). The black box indicates the area of interest depicted in the next row

Among the above-mentioned variables, most are well-known [13]. The work area is computed as the area of the complex hull of the hand movements using standard methods (Jarvis’ Algorithm [23]). The distance covered refers to the total length of the hand paths. The performance success rate is defined as the ratio of spheres intercepted over the total. However, we introduce also the new descriptors ‘Smoothness’ and ‘Total-goal directed movement’ (TGDM). Specifically, to extract information on UE motor function we introduce an original kinematic descriptor, $J(\sigma )$, to assess the patient’s movements at a specific temporal resolution, $\sigma$. This metric allows us to isolate goal-oriented movements from the hand trajectory in a certain direction, assumed to be stored over time as a function *f*(*t*). For the main dataset, we consider the left/right direction, as it is the principal axes in the movement dynamics of the ‘Spheroids’ protocol. We assume measurements are taken at discrete time points $t_i = i\Delta$ for $i = 0,1,\ldots ,T - 1$, with $\Delta$ being the timestep (for the ‘Spheroids’ dataset we have $\Delta \simeq 0.01$ s). We define the total hand displacement during goal-oriented movements $J(\sigma )$ as the difference between the actual movements and a smoothed version of the discrete movements. The smoothed hand path $f_\sigma (t)$ is obtained using a Gaussian smoothing process

3 $$\begin{aligned} f_\sigma (t_i)&= \frac{\sum _{j=0}^{T-1} f(t_j) \exp \left[ -\frac{ (t_i - t_j)^2}{2\sigma ^2}\right] }{\sum _{j=0}^{T-1} \exp \left[ -\frac{ (t_i - t_j)^2}{2\sigma ^2}\right] } \end{aligned}$$

where the parameter $\sigma$ defines how smooth the new trajectory will be, see an example in Fig. 2. Therefore $J(\sigma )$ is obtained as

4 $$\begin{aligned} J(\sigma ) = \sqrt{\frac{\sum _{i=0}^{T-1} \left[ f_\sigma (t_i) - f(t_i) \right] ^2}{T} } \, . \end{aligned}$$

**Fig. 3:**
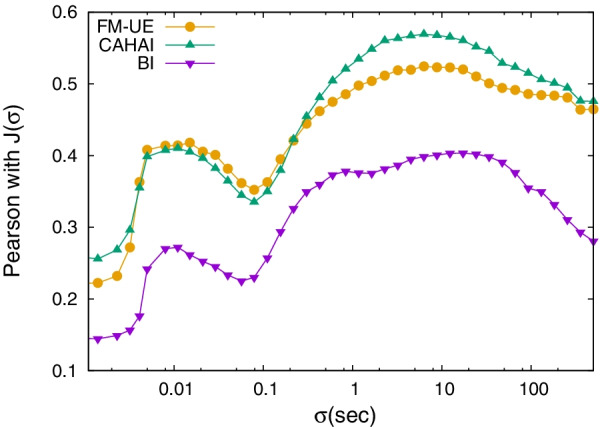
Pearson correlation coefficient between clinical scales (FM-UE, CAHAI, BI) and $J(\sigma )$ (Eq. 4) obtained for the lateral (left/right) direction of the paretic hand trajectory at different values of $\sigma$ for the ‘Spheroids’ dataset (Table 1). The high-frequency peak is at about $\sigma =0.01$ s for all three scales. The low-frequency peak is at $\sigma =8.8$ s for FM-UE, $\sigma =5.9$ s for CAHAI, and $\sigma =17$ s for BI

Following this analysis method, we derive the two new variables corresponding to the value of $J(\sigma )$ at the two peaks in the $\sigma$-dependent Pearson correlation with the clinical scales, Fig. 3: ‘Smoothness’ in correspondence to the high-frequency peak, and ’TGDM’ in correspondence to the low-frequency peak. The location of the two peaks is weakly dependent on the clinical scale considered, yet it appears to be related to the data structure: the high-frequency peak is linked to the time resolution of the data ($\Delta \simeq 0.01$ s), while the low-frequency peak is related to the typical timescale of the Spheroids protocol (i.e. a set of spheres is launched every $\sim 10$ s).

### Models description

To combine variables for estimating clinical scores of impairment and recovery, we introduce a model that allows for the presence of noise on both the variables ${\varvec{Z}}$ and the score *S*, and we hence name it a double-noise parametric model. Its generative functional form is

5 $$\begin{aligned} S({\varvec{Z}} | \varvec{\theta })&= \frac{B-A}{2} \tanh \left( \varvec{\beta } \cdot {\varvec{Z}} + \beta _0 + \sigma _1 u \right) \nonumber \\&\quad + \frac{B+A}{2} + \sigma _2 v \end{aligned}$$

where $\varvec{\theta } = \{\varvec{\beta }, \beta _0, A, B, \sigma _1, \sigma _2 \}$ are the $p+5$ model hyperparameters to be inferred: $\varvec{\beta }$ (association parameters of *p* active variables), $\beta _0$ (parametric offset), *A* and *B* (range offsets), $\sigma _1$ and $\sigma _2$ (noise strengths). The sources of noise *u* (the covariate noise) and *v* (the score noise) are both assumed to be standard normally distributed. Since $\tanh (-x) = - \tanh (x)$, we remove the resulting parameter sign ambiguity by enforcing $B \ge A$. Note that the saturation of the sigmoidal function captures the limits of the clinical scores so that the average over the noise of the estimated score *S* is constrained in the interval [*A*, *B*].

The probability of a particular score *S*, given the variables and the hyperparameters, is given by

6 $$\begin{aligned}&p(S|{\varvec{Z}}, \varvec{\theta })\\&\quad = \frac{1}{\sigma _2\sqrt{ 2\pi }} \int Dv ~ \mathrm{e}^{-\frac{1}{2 \sigma _2^2} [S- a\tanh ( \varvec{\beta } \cdot {\varvec{Z}} + \beta _0 +\sigma _1 v) -b ] ^2 } \nonumber \end{aligned}$$

with the short-hands $D v = (2 \pi )^{-\frac{1}{2}} \mathrm{e}^{-\frac{1}{2} v^2 } dv$, $a=(B-A)/2$, $b=(B+A)/2$. We use the following (improper) prior distribution over the parameters $\varvec{\theta }$:

7 $$\begin{aligned} p(\varvec{\theta }) = Z^{-1} \mathrm{e}^{-\frac{1}{2} d \varvec{\beta }^2} p(\sigma _1) \, p(\sigma _2). \end{aligned}$$

We take $p(\sigma _1) \sim \mathrm{e}^{-q/\sigma 1}$ and similar for $\sigma _2$ with *q* being a very small number, typically of the order of the accuracy of the numerical work, e.g. $q \sim 10^{-10}$. These priors guarantee that the log-likelihood function is bounded from below.

We adopt Maximum A Posteriori (MAP) inference: given the dataset ${\mathcal {D}}=\{({\varvec{Z}}_1,S_1), \ldots , ({\varvec{Z}}_n,S_n)\}$, the optimal parameters $\varvec{\theta }$ correspond to the maximum of the posterior probability or, alternatively, to the minimum of the regularised log-likelihood function:

8 $$\begin{aligned} \Omega (\varvec{\theta })&= -\sum _{i=1}^n \log \int Dz ~ \mathrm{e}^{-\frac{1}{2\sigma _2^2} [S_i - a\tanh ( \varvec{\beta } \cdot {\varvec{Z}}_i + \beta _0 +\sigma _1 z) -b ] ^2 } \nonumber \\&\quad +\frac{1}{2} d \varvec{\beta }^2 +n\log (\sigma _2) + \frac{q}{\sigma _2} + \frac{q}{\sigma _1} \, . \end{aligned}$$

The errors on the inferred parameters $\varvec{\theta }$ are estimated from the curvature of the regularised log-likelihood function at the minimum.

Two simpler models derived from Eq. 8 can be considered corresponding to having either score noise only or covariate noise only:

- *Score noise model*, $\sigma _1 = 0$. Taking the limit $\sigma _1\rightarrow 0$ in Eq. 8 gives
  9 $$\begin{aligned} \Omega _{\text {sco}}(\varvec{\theta })&=\frac{1}{2\sigma _2^2} \sum _{i = 1}^n \left[ S_i - b - a \tanh (\varvec{\beta } \cdot {\varvec{Z}}_i + \beta _0) \right] ^2 \nonumber \\&\quad + n \log (\sigma _2 ) + \frac{1}{2} d \varvec{\beta }^2 +\frac{q}{\sigma _2} , \end{aligned}$$
- *Covariate noise model*, $\sigma _2 = 0$ Taking the limit $\sigma _2\rightarrow 0$ in Eq. 8 gives
  10 $$\begin{aligned} \Omega _{\text {cov}}(\varvec{\theta })&=\frac{1}{2\sigma _1^2}\sum _{i=1}^n \Big [ \tanh ^{-1}\Big (\frac{S_i - b}{a}\Big ) - \varvec{\beta } \cdot {\varvec{Z}}_i - \beta _0 \Big ] ^2 \nonumber \\&\quad + \sum _{i=1}^n \log \big [a^2 - (S_i - b)^2\big ] +n\log \Big (\frac{\sigma _1}{a}\Big ) \nonumber \\&\quad +\frac{1}{2} d \varvec{\beta }^2 +\frac{q}{\sigma _1} \end{aligned}$$
  provided $|S_i - b|<a$ for all *i*, otherwise $\Omega _{\text {cov}}(\varvec{\theta }) = \infty$. This implies that for each *b* the minimisation over *a* is to be carried out strictly over the open interval $a>\mathrm{max}_i |S_i - b|$.

## Results

### Identification of features

**Fig. 4:**
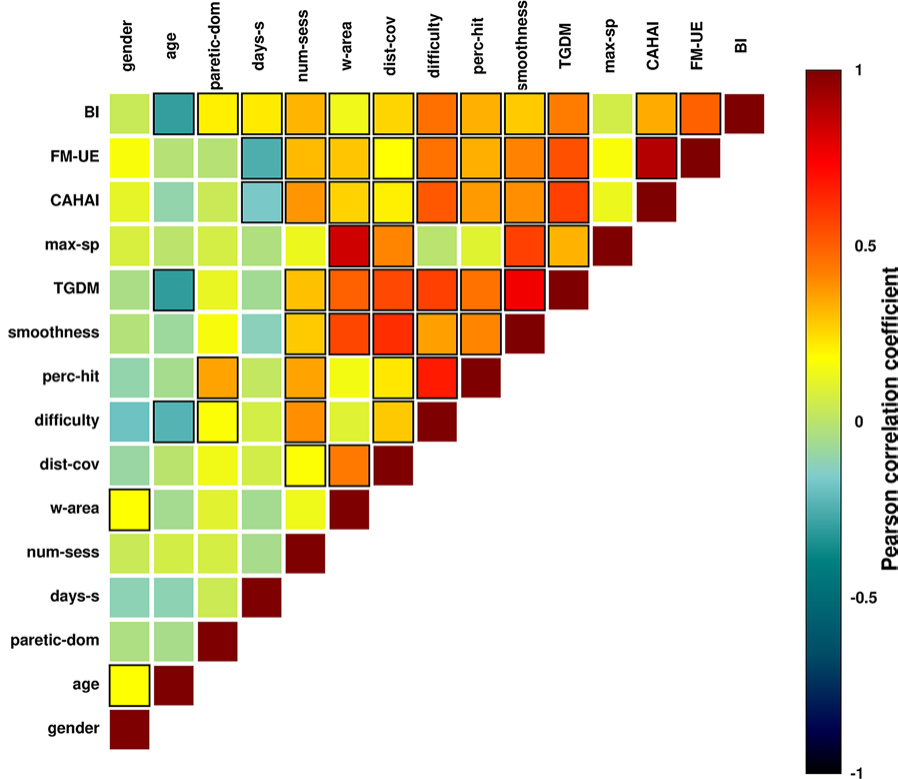
Correlogram of clinical scales and variables, Table 1. The scale indicates the value of the correlation coefficients, going from − 1 (full negative correlation) to 1 (full positive correlation). Black bordered squares indicate significant correlations ($p<0.05$)

Several of the kinematic descriptors, in particular *TGDM* and the difficulty level reached (*difficulty*) correlate highly with all clinical scales, cf. Table 1 and Fig. 4. Generally, there is a high level of consistency between correlations of the variables with the different clinical scales examined. The main exceptions are *age* and the time since stroke (*days-s*). The former does not display a relevant correlation with FM-UE and CAHAI scores but it is negatively correlated with BI ($r=-0.30$, $p < 0.0001$), while the latter correlates negatively with the FM-UE ($r=-0.25$, $p=0.00049$) and CAHAI ($r=-0.17$, $p=0.019$) scores but positively with BI ($r=0.22$, $p=0.0033$). The correlation between FM-UE and CAHAI scores is high ($r=0.89$, $p<0.0001$), while the consistency with BI is significantly lower: ($r=0.34$, $p<0.0001$ with FM-UE; $r=0.5$, $p<0.0001$ with CAHAI). Generally, the correlations of the kinematic descriptors are higher with FM-UE and CAHAI than with BI.

All kinematic variables show consistent inter-variables correlation, cf. Fig. 4. In particular, the maximum speed achieved during reaching (*max-sp*) correlates highly with the work area (*w-area*) ($r=0.76$, $p<0.0001$). The variable *age* correlates negatively with *TGDM* ($r=-0.31$, $p < 0.0001$) and *difficulty* ($r=-0.24$, $p = .00098$), while it is uncorrelated with FM-UE and CAHAI scores, but not to BI ($r=-0.30$, $p < 0.0001$). This suggests that age-related technology proficiency may affect the kinematic descriptors. Nevertheless, this effect is relatively weak, i.e., the correlations of *difficulty* and *TGDM* with the clinical scores are significantly higher than the ones with *age*.

To better understand the meaning of the two variables obtained with the smoothing techniques (*smoothness* and *TGDM*), we also extract finite time-windowed variables for comparison (64 s for the range variable; 14 s for the speed). Specifically, we compute the maximum range of movement (left/right direction) within overlapping time windows of size $\sigma$, where $\sigma$ is again fixed by the condition of maximum correlation with the clinical scale of interest, and we average the measurements across all possible windows along the whole RGS session. The resulting values show a very high correlation with the *TGDM* variable ($r=0.98$, $p<0.01$) and show very similar Pearson coefficients with the FM-UE ($r=0.54$, $p<0.01$), CAHAI ($r=0.57$, $p<0.01$) and BI ($r=0.44$, $p<0.01$) clinical scales. These results suggest that the *TGDM* is capturing information about the typical range of movement associated with the scenario events occurring within relevant time windows (i.e. about 10 seconds in the *Spheroids* scenario). Following the same method, we extract time-windowed maximum reaching speed. The resulting values show a very high correlation with the smoothness variable ($r=0.87$, $p<0.01$) together with very similar Pearson coefficients with the FM-UE ($r=0.42$, $p<0.01$), CAHAI ($r=0.39$, $p<0.01$) and BI ($r=0.21$, $p<0.01$) clinical scales. These results suggest that smoothness is linked to the ability of the patient to perform fast movements to complete the RGS tasks.

### Convergent validity: estimating impairment

We found that the three models implemented (double noise model, score noise model, covariate noise model) offer comparable performance in estimating the FM-UE scale on the dataset in Table 1. The typical error in the final estimate is of order $\sim 10$, while the inferred noise score error $\sigma _2$ is typically $\sim 0.1$. This result supports that the score noise has a low impact, therefore we select the covariate noise model to decrease the number of parameters to be inferred.

**Table 3:** The association parameters $\beta$ of the optimal variable set for estimating FM-UE scores and FM-UE change ($\Delta {\text {FM-UE}}$), *Spheroids* protocol

Covariate	$\beta ({\text {FM-UE}})$	$\beta (\Delta {\text {FM-UE}})$
Difficulty	0.186(0.051)	0.194(0.062)
TGDM	0.049(0.087)	0.184(0.090)
Diff. Distance covered	0.027(0.044)	− 0.220(0.080)
Diff. TGDM	− 0.197(0.057)	− 0.043(0.070)
Log. work area	0.073(0.059)	− 0.108(0.064)
Log. smoothness	0.086(0.079)	− 0.070(0.078)

Note that for FM-UE change the value of a variable refers to the difference between the two sessions. The values listed here refer to the normalised variables, so that the values of the different $\beta$s are directly comparable. Estimated standard deviations in parentheses

**Fig. 5:**
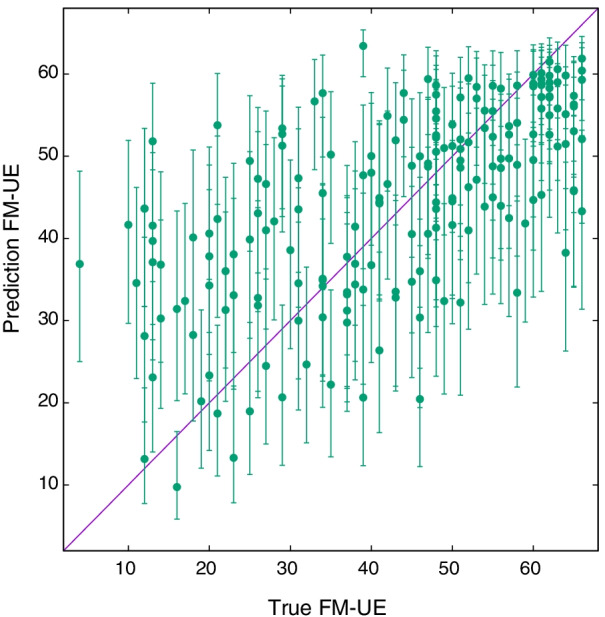
True ${\text {FM-UE}}$ versus predicted ${\text {FM-UE}}$ for the 191 samples of Table 1 (*Spheroids* scenario), using the covariate noise model with association parameters given in Table 3

The most relevant variables for the estimate of the FM-UE score are *difficulty* and *Diff. TGDM* (difference in *TGDM* between the non-paretic arm and the paretic arm). The total number of active variables is 6 and they are shown in Table 3. We have enforced the presence of *Diff. distance covered* in the active variable set since it is relevant in the estimate of clinical change, Tables 2 and 6. Note that the resulting active variable set does not contain the patient’s baseline variables and the estimation of scores on clinical scales are solely obtained from RGS-derived data. This also means that estimates made by this model for different RGS sessions of the same patient are considered independent measurements. In total, we use 10 parameters (6 association parameters and 4 hyperparameters) inferred from the 191 sessions.

The hyperparameters of the model that predicts FM-UE are given by the following mean values and standard deviations: $a=32.72 (0.85)$, $b=34.55 (0.73)$, $\sigma _1=0.551 (0.043)$ and $\beta _0 = 0.370 (0.051)$. Using LOOCV, we obtain that for the active variable set Table 3 the accuracy on the training set is 0.755 while the accuracy on the validation set is 0.746.

Eventually, we compare the true and predicted score values, shown in Fig. 5. The model predicts the FM-UE score with an average error of $E_\text {FM} \sim 12.8$, Pearson *r* true-predicted of 0.63, and a coefficient of determination $R^2 = 0.38$. These values are intermediate between an estimation based on simple rescaling of CAHAI and BI scores, Eqs. 1,2.

To further exemplify the features of the above model, we compare with the simple linear model $S = \varvec{\beta } \cdot {\varvec{Z}} + \beta _0 + \sigma u$ with the same covariates as in Table 3. The inferred values of $\beta$ for normalised variables are in this case (same order as in Table 3) 2.32(0.71), 1.86(0.81), $-0.84 (0.72)$, $-2.58 (0.74)$, 1.27(0.75), 1.46(0.79) with $\beta _0 = 42.98 (0.66)$ and $\sigma = 12.90 (0.93)$. The linear model predicts the FM-UE scores with an average error of $E_\text {FM} \sim 17.3$, Pearson *r* true-predicted of 0.60, and a coefficient of determination $R^2 = 0.14$. Interestingly, the range of estimated FM scores in the dataset is now [16, 84], with 46 scores (over 191) estimated above 66. This result exemplifies the problem of mapping unbounded variables on a finite range and illustrates the need for a nonlinear function as in Eq. 5.

### Robustness: test–retest reliability

**Fig. 6:**
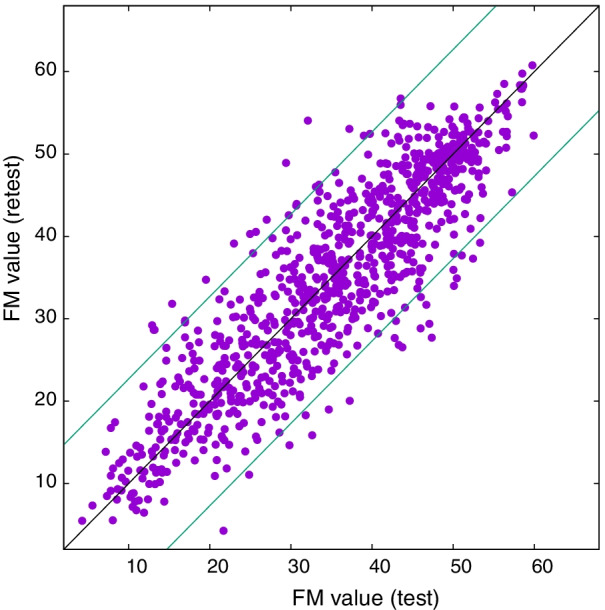
Estimate of the FM-UE scores of the first session (test) vs estimate of the FM-UE scores of second session (retest) for unseen 921 couples of RGS sessions. Each couple is recorded from a same patient within 48 hours. The estimates are obtained using the covariate noise model with association parameters given in Table 3

In Fig. 6 we show the FM-UE estimations obtained by the model for the test and retest sets. The ICC between the ‘test’ and ‘retest’ is 0.89, which is at the high end of the ‘Good’ agreement measure according to the Koo and Li guidelines [24]. In Fig. 6 we also show the interval defined by the standard error of the regression $E \simeq 12.7$. We measure an average retest error $\sqrt{\sum _i (S_i^\text {test}-S_i^\text {retest})^2/N}$ equal to 5.9. Finally, we estimate a minimally detectable change (MDC) [25] of 11.6 points.

These results support the internal consistency of this assessment method to estimate the clinical scores of the patients.

### External validity: estimating the change in impairment

We observe that the Pearson correlation between change in FM-UE ($\Delta {\text {FM-UE}}$) and change in CAHAI ($\Delta \text {CAHAI}$) is $r=0.68$, Pearson between $\Delta {\text {FM-UE}}$ and change in BI ($\Delta \text {BI}$) is $r=0.67$, while the Pearson correlation between $\Delta \text {CAHAI}$ and $\Delta \text {BI}$ is $r=0.72$.

**Fig. 7:**
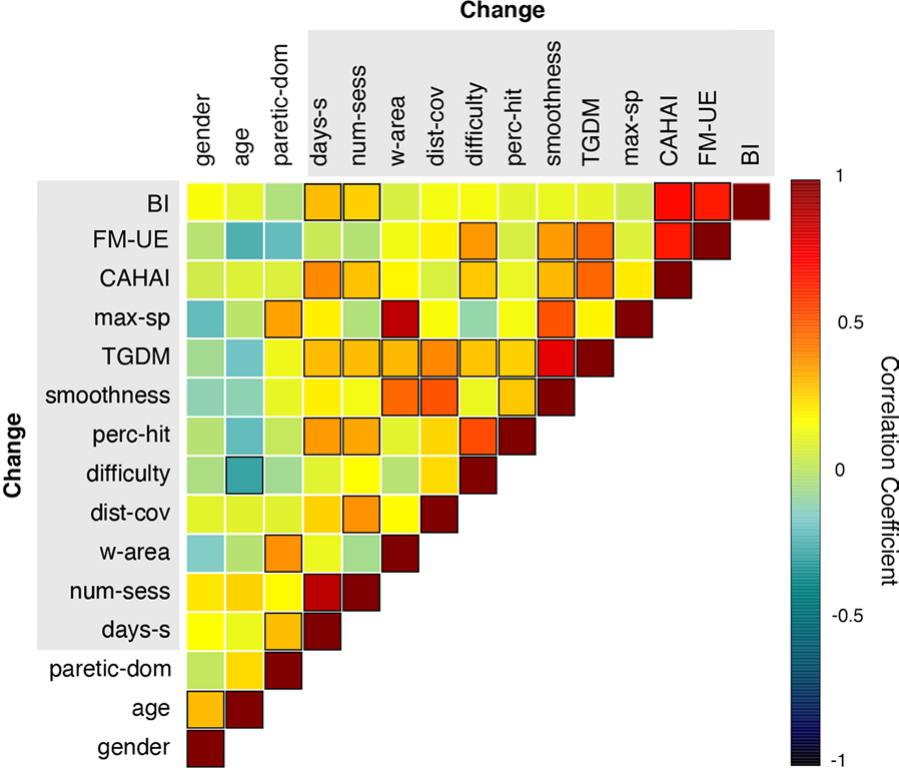
Correlogram of change of clinical scale and change in variables between couples of sessions of the same patient, Table 2. The scale indicates the value of the correlation coefficients, going from $-1$ (full negative correlation) to 1 (full positive correlation). Black bordered squares indicate significant correlations ($p<0.05$)

For each sample, we consider now as variables the change (between the two sessions) of the original variables. By comparing it to a randomised outcome distribution, we identify a threshold of $r \sim 0.14$ for the Pearson correlations variable-score. Several of the variables correlate highly with the change in all three clinical scores, cf. Tables 2 and  6 in the Appendix. The highest correlated variable is *Change in TGDM* ($r=0.48$, $p=0.00024$ with $\Delta {\text {FM-UE}}$; $r=0.55$, $p<0.0001$ with $\Delta \text {CAHAI}$; $r=0.49$, $p=0.0011$ with $\Delta \text {BI}$). Note that, in comparison to estimating a single session’s score, the variables *age* and *chronic* correlate more with the outcome when predicting the score change. The initial scores (the clinical scores at the first session) have a high correlation with change because of ceiling effects. In Fig. 7 we show the correlogram of all the variables and clinical scales. We observe that generally the correlations between kinematic descriptors in a single session (Fig. 4) are preserved also when considering the change between sessions; for example, the highest inter-variables correlation is for *Change in w-area* and *Change in max-sp* at ($r=0.87$, $p<0.0001$).

The association parameters of the model that estimates $\Delta {{\text {FM-UE}}}$ are shown in Table 3. The corresponding model hyperparameters are $a=23.3 (3.3)$, $b=20.9 (3.2)$, $\sigma _1=0.430 (0.060)$, and $\beta _0 = -0.68 (0.12)$.

**Fig. 8:**
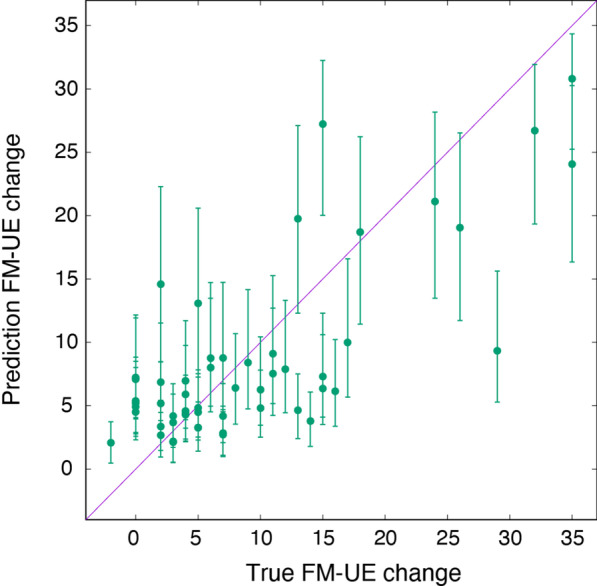
True versus predicted $\Delta {\text {FM-UE}}$ for 54 data points of recovery dataset (Table 2), using the covariate noise model with association parameters given in Table 4

We compare the true and predicted $\Delta {\text {FM-UE}}$ in Fig. 8. The Pearson correlation between true $\Delta {\text {FM-UE}}$ and predicted $\Delta {\text {FM-UE}}$ is 0.76. The value of the coefficient of determination $R^2$ is 0.57. These results show that the model has a good external validity to clinical change with a precision comparable to the one obtained for the cross-sectional FM-UE score.

### Sensitivity: estimating recovery

We obtain that the model correctly predicts recovery in 36 over 38 of the cases with $\Delta {\text {FM-UE}} \ge 4$, indicating a TPR of 95%.

### Task generalisation

For the task generalisation analysis, we consider a dataset composed of 37 RGS sessions from 19 hemiparetic participants that trained in a rehabilitation protocol derived from *Whac-A-Mole* [18].

Unlike the *Spheroids* protocol, the gameplay of *Whac-A-Mole* requires movements on the full 2d plane. In response, we utilise the smoothing technique in both cardinal axes of the task. i.e. front/back and left/right directions. Pearson correlations between the clinical scales and the variable $J(\sigma )$ reveal a similar pattern to the one observed in the Spheroids scenario, with a peak of the Pearson coefficients at about 1*s* corresponding to the variable *TGDM* in each direction (Fig. 13 in Appendix). The location of the main peak is again close to the typical timescale of the protocol. For the FM-UE score, the highest Pearson coefficient is observed in the frontal direction ($r=0.54$ for $\sigma = 1.3 s$); the lateral hand displacement peak is ($r=0.50$ at $\sigma = 1.1 s$).

When predicting clinical scales, we use now only 2 active variables in order to limit overfitting: the variables TGDMfb (Total-goal directed movement for front/back direction) and TGDMlr (Total-goal directed movement for left/right direction). We then infer 6 parameters (2 association parameters + 4 hyperparameters) from the 37 RGS sessions. The two association parameters are (for normalised variables) $\beta _\text {TGDMfb} = 0.15(0.13)$ and $\beta _\text {TGDMlr} = 0.18(0.14)$. The hyperparameters of the model that predicts FM-UE for the ‘Whac-A-Mole’ scenario are given by $a=31.9 (3.5)$, $b=31.0 (2.5)$, $\sigma _1=0.49 (0.11)$, and $\beta _0 = 0.21 (0.13)$.

**Fig. 9:**
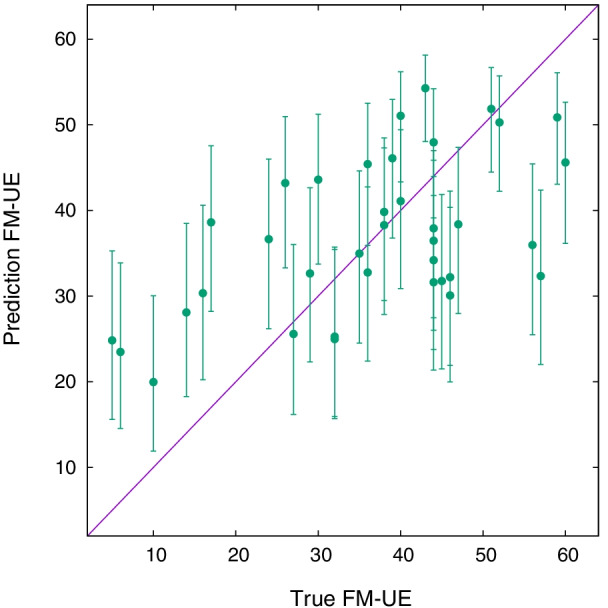
True versus predicted ${\text {FM-UE}}$ for 37 samples of the *Whac-A-Mole* dataset, using the covariate noise model with two active variables (TGDMfb and TGDMlr)

The FM-UE estimates are shown against the true values in Fig. 9. The Pearson correlation between true FM-UE and predicted FM-UE is 0.63, the average error is $E \sim 11.2$, and the value of the coefficient of determination $R^2$ is 0.39.

### Generalisation to CAHAI and BI

**Fig. 10:**
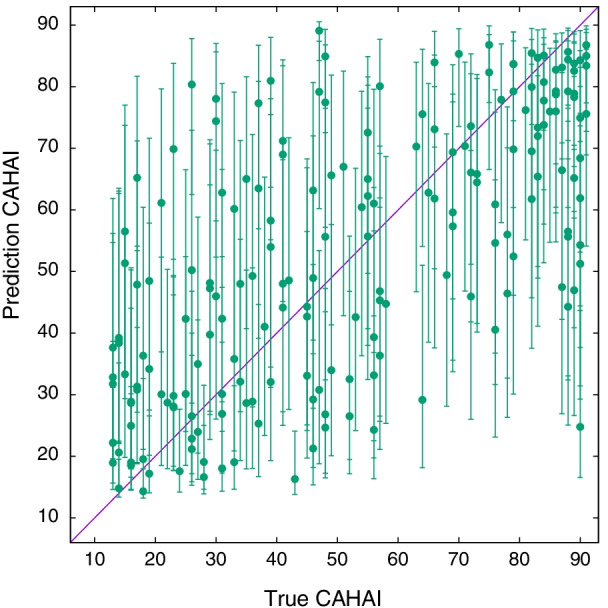
True versus predicted CAHAI for 191 data points of *Spheroids* dataset (Table 1), using the covariate noise model with association parameters given in Table 4

Overall, the CAHAI scale has similar properties to FM-UE in relation to the kinematic descriptors, Fig. 4. To stress the generalisation potential of the model, we can then adopt the same model introduced in Table 3 for estimating the FM-UE and CAHAI scores. The association parameters for CAHAI are reported in Table 4. The most important variables are *difficulty* and *TGDM*. The hyperparameters of the covariate noise model that predicts CAHAI scores are $a=39.158 (0.099)$, $b=51.962 (0.079)$, $\sigma _1=0.953 (0.064)$, and $\beta _0 = 0.0319 (0.071)$. The predicted scores are plotted against the true CAHAI values in Fig. 10. The model predicts the CAHAI score with an average error of $E_\text {CAHAI} \sim 20.1$, Pearson *r* true-predicted of 0.66, and a coefficient of determination $R^2 = 0.40$. This accuracy is close to what we obtained for estimating the FM-UE score, Fig. 5.

**Table 4:** The association parameters $\beta$ for estimating the CAHAI score, *Spheroids* scenario

Covariate	$\beta (\text {CAHAI})$
Difficulty	0.333 (0.087)
TGDM	0.36 (0.15)
Diff. Distance covered	0.016 (0.088)
Diff. TGDM	− 0.187 (0.098)
Log. work area	0.09 (0.10)
Log. smoothness	− 0.04 (0.14)

The active variables are the same as in Table 3. The values refer to the normalised variables so that the values of the different $\beta$s are directly comparable.

**Fig. 11:**
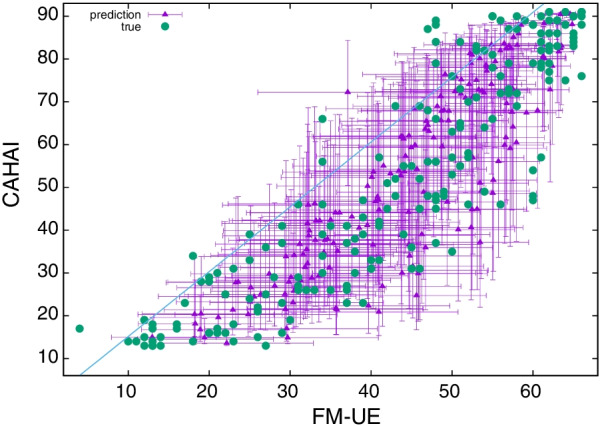
True FM-UE versus true CAHAI (green dots) and predicted FM-UE versus predicted CAHAI (purple triangles with errorbars) for 191 data points of *Spheroids* database (Table 1). We use the covariate noise model with association parameters given in Table 3 for FM-UE and Table 4 for CAHAI

**Fig. 12:**
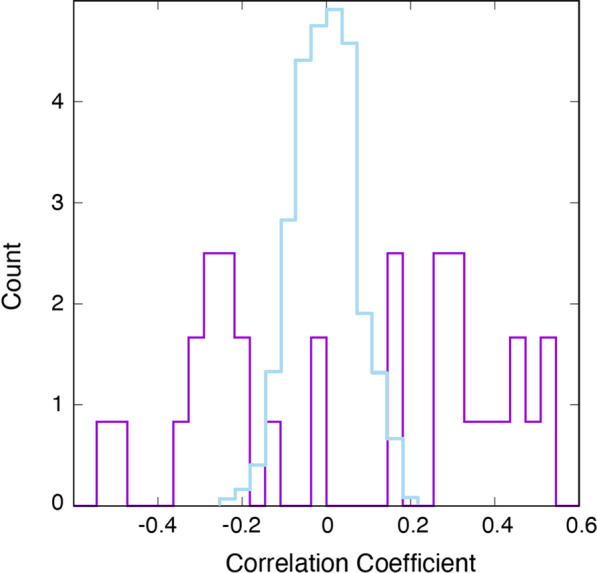
Distribution of Pearson correlation coefficients measured between the FM-UE scores and all the variables in Tables 1 and 5. The light blue line is the distribution obtained for random outcomes

**Fig. 13:**
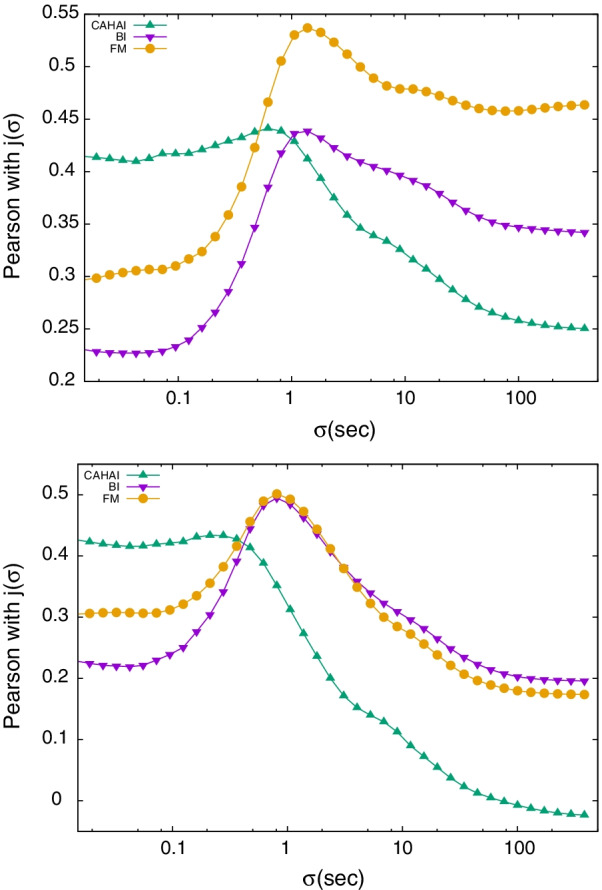
Pearson correlation coefficient between clinical scales (FM-UE, CAHAI, BI) and $J(\sigma )$ Eq. 4 in the front/back direction (top) and left/right direction (bottom) at different values of the timescale $\sigma$ on dataset *Whac-A-Mole* ($N=37$, Sec. Task Generalisation). For the FM-UE score, the peaks are at ($r=0.54$ for $\sigma = 1.3 s$) for the frontal direction and at ($r=0.50$ at $\sigma = 1.1 s$) for the lateral direction. For the CAHAI score, the peaks are at ($r=0.44$ for $\sigma = 0.78 s$) for the frontal direction and at ($r=0.43$ at $\sigma = 0.31 s$) for the lateral direction. For the BI, the peaks are at ($r=0.44$ for $\sigma = 1.2 s$) for the frontal direction and at ($r=0.49$ at $\sigma = 1.1 s$) for the lateral direction

In Fig. 11 we compare FM-UE and CAHAI, both for the true scores and the predicted scores. We note that the relationship between FM and CAHAI is generally well preserved in the estimates; for example, the Pearson between FM-UE and CAHAI scores is $r=0.89$ for true values and $r=0.88$ for estimates. The fact that the variability in the true FM-UE vs true CAHAI is seemingly comparable to the one in the model, reinforces the idea that the precision we achieve is similar to the one of estimating FM-UE directly from CAHAI, as we estimated using Eq. 1.

Finally, we observe that the model that predicts FM-UE and CAHAI scores does not work well for the BI. Most kinematics variables have a significantly smaller correlation with the BI (in particular *work area*, *TGDM*, *smoothness*) while baseline information and clinical history of the patient are comparatively more relevant (for example the patient’s age, Table 1 and Fig. 4). The active variable set for BI is composed of 5 variables ($\beta$ values for normalised variables): *age* ($\beta =-0.21(0.15)$), *sessions completed so far* ($\beta =0.24(0.19)$), *difficulty* ($\beta =1.21(0.72)$), *Log. time since stroke* ($\beta =0.14(0.14)$), *Log. Difficulty* ($\beta =-0.91(0.70)$). Using a double noise model, Eq. 8, we infer the hyperparameters $a=51.56 (0.10)$, $b=49.22 (0.12)$, $\sigma _1=0.5948 (0.0072)$, $\sigma _2=0.178 (0.027)$, and $\beta _0 = 1.025 (0.011)$. Comparing true and predicted BI scores, we measure an average standard error of $E_{\text {BI}} \sim 16.8$, a Pearson correlation true-prediction of 0.62, and a coefficient of determination $R^2$ of 0.35. This accuracy is comparable to the one achieved by the models for FM-UE and CAHAI scores. Nevertheless, the dataset Table 1 is very unbalanced towards high BI scores (mean score 80, with only 3 samples with a score below 25), so may not generalise well to homogeneous unseen BI data (i.e., the precision for low scores may be relatively poor).

## Discussion and conclusion

Our understanding of post-stroke motor recovery depends on our capacity to evaluate and characterise impairment and disability. Current standardised assessment methods are mostly subjective and present relevant unsystematic variability due to differences in the evaluators’ training, lack of systematicity in the administration of the assessments, and often are excessively focused on one single aspect of the impairment and/or disability.

Different rehabilitation approaches show a preference for using (and even targeting) specific assessment methods for the evaluation of their therapeutic efficacy, and often these methods have been developed by the same team of authors. For example, the effectiveness of Constraint-Induced Movement Therapy [26] is usually evaluated using the Wolf Motor Function Test [27] and the Motor Analog Scale [28], while the effectivity of occupational therapy has been frequently assessed using the Barthel Index [29] and the Functional Independence Measure [30]. There is an urgent need to establish alternative methods for a common evaluation protocol and characterisation of the hemiparesis phenotype, thus allowing us to identify specific impairment features that could advance our understanding of the recovery dynamics and guide the design of effective rehabilitation therapies. In pursuing this objective, we have conducted a careful analysis of the kinematic data from the upper-extremities of 191 individuals with post-stroke hemiparesis, and we have constructed a model for estimating impairment and recovery. Our results reveal a new digital biomarker of upper-limbs motor impairment, the Total Goal-Directed Movement (TGDM), which relates to the patients’ range of motion during the execution of meaningful goal-oriented reaching movements. The *TGDM* strongly correlates with the level of impairment captured by the FM-UE and the level of disability captured by the CAHAI, and also carries relevant information about the patients’ progress, showing a high correlations with the magnitudes of improvement and deterioration estimated by both scales. The model presents high external validity of impairment estimates ($R^2$: 0.38), robustness (test–retest reliability) (ICC: 0.89), external validity of recovery estimates ($R^2$: 0.57), sensitivity (TPR: 95%) and task generalisation ($R^2$: 0.39). Despite the high heterogeneity of the sample and the high level of noise of the selected kinematic parameters, the model’s accuracy to estimate the FM-UE is comparable to other standardised clinical scales, such as CAHAI. These results are especially interesting given the currently limited evidence about the external validity of the change of kinematic outcome measures of reaching performance in people with hemiparesis after stroke [31]. According to a recent systematic review on the clinimetric properties of kinematic upper limb assessments [13], only two papers captured external validity of change (i.e., ability to capture longitudinal changes in the measured construct), and just nine parameters showed enough evidence to estimate recovery (i.e., number of velocity peaks, trunk displacement, task/movement time). The quality of evidence however was very low for all metrics. Further, our results outperform previous methods to estimate the level of impairment [15, 16] in two fundamental aspects: (1) it shows robustness to compensation and is resistant to using explicit strategies to boost performance, and (2) it exclusively relies on metrics collected under unsupervised rehabilitation sessions. Although current recommendations point out that wearables with integrated Inertial Measurement Units and vision-based tracking systems are insufficient to measure the quality of movement and improvement in motor function, our findings, together with the growing evidence supporting distance travelled as an accurate and responsive digital biomarker of recovery [32], suggest the opposite and advocate for further studies to clarify the limitations of these estimations, in particular regarding the responsiveness.

Although these results support that our method is able to capture improved sensorimotor control during gameplay (i.e., motor synergies), we cannot discard the contribution of task-related learning processes to the overall change measured. In addition, it is important to notice that the RGS setup did not provide antigravity support, thus the different capabilities of the participants to elevate the elbow and wrist may explain part of the unsystematic variability we observe in the model’s performance.

The relevance of our results is emphasised by their consistency across clinimetric properties and by their generalisation potential, relying on a large and heterogeneous dataset of patients at different stages post-stroke. Notice however that its generalisation potential has been evaluated in similar setups using vision-based systems (camera- or depth-sensor-based) tracking the joints of the upper limbs during the performance of planar reaching arm movements. We believe that the applicability of the *TGDM* and its derived models to evaluate impairment and motor recovery is promising for a number of reasons: (1) it can be derived from unimanual displacements executed in the horizontal plane, (2) it does generalise to other tasks involving two-dimensional horizontal reaching movements towards targets, and (3) it can be estimated during unsupervised motor training. Our results provide an example of how digital biomarkers of motor deficits derived from behavioural data could guide the design of automated assessment platforms for a continuous and remote monitoring of impairment.

## Data Availability

The datasets used and analysed in this study are available from the corresponding author on reasonable request.

## Appendix

In Table 5 we report the descriptive statistics of the secondary variables (functions of primary variables in Table 1) for dataset *Spheroids*.

**Table 5:** Characteristics of the secondary variables for the 191 samples composing the main dataset (obtained from first-order variables, Table 1)

Variables	Range [min, max]	Mean	SD	*r*(FM-UE)	*r*(CAHAI)	*r*(BI)
15. Chronic	Yes/no	57/134	–	− 0.25	− 0.17	0.14
${\texttt {<}}$Subacute${\texttt {>}}$	Yes/no	74/117	–	–	–	–
16. Acute	Yes/no	60/131	–	0.32	0.18	− 0.15
17. Diff. work area ($m^2$*)*	[− 1.3, 1.6]	0.25	0.50	− 0.31	− 0.29	(− **0.069**)
18. Diff. distance covered (*m**)*	[− 160, 120]	11	34	− 0.32	− 0.26	− 0.12
19. Diff. performance (% success)	[− 0.22, 0.53]	0.087	0.13	− 0.26	− 0.22	− 0.16
20. Diff. maximum reaching speed (*m*/*s**)*	[− 61, 98]	8.1	23	− 0.22	− 0.22	− 0.15
21. Diff. difficulty level reached	[− 0.47,0.63]	0.11	0.19	− 0.22	− 0.15	(− **0.058**)
22. Diff. smoothness (mm*)*	[− 2.6, 4.4]	0.34	0.75	− 0.29	− 0.28	− 0.20
23. Diff. TGDM (m*)*	[− 0.035, 0.084]	0.015	0.021	− 0.52	− 0.47	− 0.24
24. Log. time since stroke (days)	[1.6, 8.0]	4.8	1.7	− 0.24	(− **0.076**)	0.36
25. Log. sessions completed so far	[0.0, 3.9]	1.8	0.9	0.29	0.38	0.42
26. Log. work area	[− 4.3, 0.62]	− 1.3	0.9	0.40	0.38	0.19
27. Log. distance covered	[0.92, 5.5]	3.8	0.7	0.26	0.32	0.34
28. Log. maximum reaching speed	[1.0, 4.5]	2.6	0.73	0.26	0.20	(**0.045**)
29. Log. difficulty level reached	[− 0.16, 0.60]	0.25	0.14	0.44	0.50	0.45
30. Log. smoothness	[− 1.7, 1.4]	0.012	0.48	0.48	0.46	0.33
31. Log. TGDM	[− 4.7, − 2.1]	− 2.9	0.48	0.52	0.56	0.43

The *r* columns refer to the Pearson correlation coefficients with the FM-UE, CAHAI, and BI clinical scales, respectively. Correlations below the threshold $r \sim 0.081$ (Fig. 12 in Appendix) are in parenthesis. From the time since stroke, we obtain the categories Acute (5–90 days), Sub-acute (3–12 months), and Chronic (over 1 year). The variables obtained directly from the RGS system log files are in Italic type. The *Diff.* variables are obtained as the difference between the value observed for the less affected arm and the value for the more affected one. The *Log.* variables are obtained as the natural logarithm of the corresponding first-order variables

The distribution of the Pearson correlation coefficients of the FM-UE score with all the variables in Tables 1 and 5 is shown in Fig. 12. This is compared with the distribution obtained with the randomised outcome, whose standard deviation is $r \simeq 0.081$.

In Table 6 we report the descriptive statistics of the secondary variables (functions of primary variables in Table 2) for the recovery dataset (change of variables and clinical scales between two RGS sessions of the same patient with a delay of at least 16 days).

**Table 6:** Characteristics of the secondary variables for the 54 samples composing the recovery dataset (obtained from first-order variables, Table 2)

Variables	Range [min,max]	Mean	SD	$r(\Delta {\text {FM-UE}})$	$r(\Delta \text {CAHAI})$	$r(\Delta \text {BI})$
15. Chronic	Yes/no	10/44	–	− 0.30	− 0.44	− 0.38
${\texttt {<}}$Subacute${\texttt {>}}$	Yes/no	10/44	–	–	–	–
16. Acute	yes/no	34/20	–	0.19	0.44	0.53
17. Change in diff. work area (m*)*	[− 1.5, 1.8]	− 0.13	0.67	− 0.30	(− **0.060**)	− 0.24
18. Change in diff. distance covered (m)	[− 94, 150]	3.1	40	− 0.45	0.23	− 0.30
19. Change in diff. performance (% success)	[− 0.22, 0.53]	0.087	0.13	0.18	(**0.10**)	0.17
20. Change in diff. maximum reaching speed (m/s*)*	[− 76, 71]	− 5.1	30	− 0.28	− 0.16	− 0.32
21. Change in diff. difficulty	[− 0.46, 0.62]	0.0095	0.21	(**0.064**)	(**0.12**)	0.14
22. Change in diff. smoothness (mm*)*	[− 2.7, 1.4]	− 0.17	0.85	− 0.41	− 0.26	− 0.47
23. Change in diff. TGDM (m*)*	[− 0.12, 0.088]	− 0.0050	0.040	− 0.51	− 0.43	− 0.56
24. Log. time since stroke (at first)	[0.41, 1.2]	0.73	0.16	− 0.36	− 0.37	− 0.40
25. Log. sessions completed so far (at first)	[0, 1.2]	0.52	0.39	− 0.55	− 0.36	− 0.55
26. Change in Log. work area	[− 6.4, 1.5]	− 0.79	1.6	(**0.062**)	(**0.057**)	(**0.11**)
27. Change in Log. distance covered	[− 0.90, 5.6]	3.0	2.0	0.18	0.16	0.17
28. Change in Log. maximum reaching speed	[− 1.8, 5.6]	1.4	2.3	0.15	0.30	0.17
29. Change in Log. difficulty	[− 3.8, 0]	− 1.4	0.90	(**0.064**)	(**0.13**)	(**0.024**)
30. Change in Log. smoothness	[− 2.0, 3.0]	0.63	1.1	0.31	0.32	0.37
31. Change in Log. TGDM	[− 10, 0.5]	− 1.4	1.8	(**0.035**)	0.17	(**0.037**)

We select couple of sessions of the same patient with a delay of at least 16 days from the main dataset, Table 1. The *r* columns refer to the Pearson correlation coefficients with the FM-UE, CAHAI, and BI clinical scales, respectively. Correlations below the threshold $r \sim 0.14$ are in parenthesis. The variables obtained directly from RGS system log files are in Italic type. The *Diff.* variables are obtained as the difference between the value observed for the less affected arm and the value for the more affected one. The *Log.* variables are obtained as the natural logarithm of the corresponding first-order variables

In Fig. 13 we show the Pearson’s *r* between $J(\sigma )$ Eq. 4 and the clinical scales as a function of the timescale $\sigma$ for the database *Whac-A-Mole*, Sec. Task Generalisation. This is a dataset composed of 37 RGS sessions with a limited range of the clinical scales: FM-UE range of observations [5, 60], mean 37, SD 14; CAHAI range of observations [7, 49], mean 32, SD 15; BI range of observations [48, 100], mean 85, SD 13. The *Whac-A-Mole* scenario requires movements in all 2D place (unlike *Spheroids* scenario, Table 1), so we define two independent $J(\sigma )$: one associated with the front/back trajectory and one associated with the lateral (left/right) trajectory. In Fig. 13 we show that in both directions there is a peak in the Pearson’s *r* at about $\sigma = 1$ s for all three clinical scales. In correspondence with these two peaks, we define two variables: TGDMfb (Total-goal directed movement in front/back direction) and TGDMlr (Total-goal directed movement in the left-right direction). We stress that the timescale of the peak is roughly equivalent to the timescale of the gameplay of the *Whac-A-Mole* scenario. Finally, we note that for the same analysis done in the *Spheroids* scenario we observe two clear peaks in the lateral direction (Fig. 3). In *Whac-A-Mole* instead, the high-frequency peak is not clearly visible. One factor that may affect this difference is that the gameplay of *Whac-A-Mole* is faster than *Spheroids* (roughly 1 s instead of 10 s) so that the separation between the two potential peaks is smaller. Other factors that may influence the absence of the second peak are the fact that the gameplay is 2D (so that the speed in one direction is less informative) or the inadequate time-resolution of the camera (different from the one used for *Spheroids*).

## Abbreviations

RGS: Rehabilitation gaming system
FM-UE: Fugl-Meyer Assessment for Upper Extremities
CAHAI: Chedoke Arm and Hand Activity Inventory
BI: Barthel Index
SD: Standard deviation
ICC: Intraclass correlation coefficient
UE: Upper extremities
TGDM: Total-goal directed movement
max-sp: (Figs. 4, 7) maximum reaching speed
perc-hit: (Figs. 4, 7) performance (percentage hit)
dist-cov: (Figs. 4, 7) distance covered
w-area: (Figs. 4, 7) work area
num-sess: (Figs. 4, 7) sessions completed so far
days-s: (Figs. 4, 7) time since stroke (days)
paretic-dom: (Figs. 4, 7) dominant side more affected
MDC: Minimal detectable change
TPR: True positive rate
